# Role of l-carnitine and oleate in myogenic differentiation: implications for myofiber regeneration

**DOI:** 10.20463/jenb.2018.0015

**Published:** 2018-06-30

**Authors:** Hojun Lee, Jae-Young Lim, Seung-Jun Choi

**Affiliations:** 1 Department of Sports and Health Science, Kyungsung University, Busan Republic of Korea; 2 Department of Rehabilitation Medicine, Seoul National University Bundang Hospital, Seongnam Republic of Korea

**Keywords:** L-carnitine, oleate, myoblast, myotube, myogenic differentiation

## Abstract

**[Purpose]:**

Myogenic progenitors play a critical role in injury-induced myofiber regeneration. The purpose of this study was to characterize the effects of oleate and L-carnitine on the overall behavior of proliferating myogenic progenitors (myoblasts) and its link to the mitochondrial biogenic process.

**[Methods]:**

C2C12 myoblasts were cultured either with no treatment, oleate, L-carnitine, or their mixture. Proliferating myoblasts were investigated under a phase-contrast microscope. Myonuclei and myosin heavy chain were stained with DAPI and MF20 antibody, respectively, in differentiated myotubes and visualized under florescence microscopy. Mitochondrial biogenic markers and porin were assessed by qRT-PCR or immunoblotting.

**[Results]:**

Increased proliferation rate was observed in myoblasts conditioned with oleate or a mixture of oleate and L-carnitine in contrast to that in non-treated (NT) and L-carnitine-treated myoblasts. Myoblast viability was not statistically different among all tested groups. Fusion index and myotube width were greater in oleate- or L-carnitine-conditioned myotubes than those in NT myotubes, with the greatest effect seen in myotubes conditioned with the mixture. The gene expressions of Pgc1-α, Nrf1, and Tfam were the greatest in myotubes conditioned with the mixture, whereas the level of Ncor1 expression was lower compared to those of the other groups. Protein level of porin was the greatest in myotubes conditioned with the mixture, followed by that of individually treated myotubes with oleate and L-carnitine.

**[Conclusion]:**

These results provide a critical piece of cellular evidence that combined treatment of oleate and L-carnitine could serve as a potential therapeutic strategy to facilitate biological activation of myogenic progenitors.

## INTRODUCTION

It has been well documented that the skeletal muscle, the largest tissue of the body, has potential to regenerate injured myofibers^1^. Due to the dynamic locomotive traits of the skeletal muscle, myofiber damage is the most prevalent injury in the fields of sports and exercise^2^. Skeletal muscle regeneration is orchestrated via a series of cellular events^3^. Although recent evidence implied that pericytes and interstitial cells outside the basal lamina possess a certain level of myogenic potency^4^, there is consensus that skeletal muscle regeneration is primarily modulated by the activities of myogenic progenitors called satellite cells underneath the basal lamina, which is intimately juxtaposed to sarcolemma^5^.

In the initial phase of muscle regeneration, the cellular digestion of fragmented myofibers occurs, which is followed by complete removal of cellular debris. This process is significantly coupled with a skeletal muscle inflammatory response^6^. During the inflammatory stage, monocyte-derived macrophages secrete chemotactic signaling molecules, followed by the migration of satellite cells to the sites of microscopic injury in damaged skeletal myofibers^7^, suggesting a potent role of myogenic progenitors in complete muscle regeneration.

In the field of sports and rehabilitation medicine, there has been a great deal of efforts in elucidating optimal ways to promote skeletal muscle recovery^8^. Several lines of nutritional methods have been suggested as potential noninvasive strategies without side effects^9^. L-carnitine, a derivative amino acid, is considered an essential micro-component modulating bioenergetics in myofiber via the transport of long-chain fatty acids into the mitochondrial matrix^10^, emphasizing that its availability is essential in the biochemical bioenergetics of the skeletal muscle. In line with this, it has been observed that the treatment of exogenous L-carnitine attenuates signs of tissue damage in sarcopenic muscle^11^ and promotes recovery from exercise-induced muscle injury^10^.

Multiple studies demonstrated that palmitic acid, the most abundant fatty acid, is universally toxic to a variety of tissues and cells, whereas monounsaturated fatty acids such as palmitoleic acid and oleic acid are cytoprotective^12-14^. In agreement with this, oleic acid has been documented to preserve the integrity of skeletal myotubes even with a high treatment dose^15^. Furthermore, the preincubation with oleic acid protected skeletal muscle cells from pathological conditions such as apoptosis and insulin resistance by reducing the content of detrimental fatty acid derivates and increasing carnitine palmitoyltransferase 1 (CPT1) in the mitochondria, suggesting a potential role of oleate-induced mitochondrial oxidative capacity^16^.

Given that L-carnitine is closely involved in mitochondrial beta-oxidation and that oleic acid is considered as a cytoprotective and mitochondria-stimulating unsaturated acid in the skeletal muscles, it is hypothesized that these two supplements would induce synergistic effects on myogenic progenitors’ activities. Therefore, in this study, we examined L-carnitine, oleic acid, and their mixture to primarily identify if there would be positive effects upon proliferation, migration, and myogenic differentiation of skeletal muscle progenitor cells.

## METHODS

### Cell culture

**Fig. 1.**
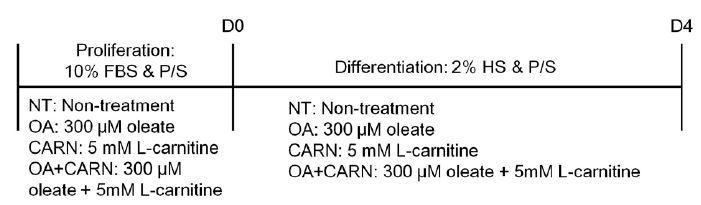
Schematic diagram of the experimental cell culture protocol. NT, non-treatment; OA, oleic acid; CARN, L-carnitine.

C2C12 myoblasts (ATCC) were seeded onto non-coated 6-well plates and maintained in Dulbecco’s modified Eagle’s medium (DMEM, Welgene, Korea) containing 10% fetal bovine serum (FBS), 100 U/mL penicillin, and 100 mg/mL streptomycin (Welgene, Korea) in a humidified atmosphere of 95% air and 5% CO2 at 37°C. For proliferation test, 5 mM L-carnitine or 300 uM oleic acid or a mixture (5 mM L-carnitine and 300 μM oleic acid) were added to the growth medium (10% FBS), based on previous studies^15^,^17^. When myoblasts were confluent (95%), the growth medium was changed to a differentiation medium (DM) supplemented with 2% horse serum (HS), 100 U/mL penicillin, and 100 mg/mL streptomycin (Welgene, Korea). DM was changed every 24 h. For the differentiation test and fusion index assay, 5 mM L-carnitine or 300 μM oleic acid or a mixture were added to DM supplemented with 2% HS and incubated for 96 h. The detailed incubation method for each experiment is described in each figure legend. Cell culture was performed a minimum of six independent times. As the fusion index is a myological indicator showing the degree of myogenic differentiation from myoblasts to myotubes, the index is calculated as the number of nuclei in myotubes divided by the total number of nuclei counted, which is expressed as a percentage. A schematic diagram of the cell culture protocol is presented in Figure 1.

### Cell counting and viability assay

Cell counting and viability assay were conducted using the trypan blue exclusion assay with minor modification^18^. C2C12 myoblasts (5 × 104/mL) were plated onto non-coated 60-mm culture dishes and grown in DMEM supplemented with 10% FBS, 100 U/mL penicillin, and 100 mg/mL streptomycin in a humidified atmosphere of 95% air and 5% CO^2^ at 37°C for 48 h. DMEM was changed on a daily basis. Following incubation, the cells were trypsinized and stained with 10 μL trypan blue on a patch of parafilm. Non-stained viable cells and stained cells were counted on a hemocytometer under a phase-contrast microscope and expressed as % NT.

### Immunocytochemistry for myosin heavy chain and myonuclei

As described^15^, myotubes were fixed and permeabilized by incubation in ice-cold methanol (Sigma-Aldrich) for 10 min. After rehydration in DPBS three times, the myotubes were incubated in blocking solution including 2% BSA for 30 min at room temperature, followed by incubation in 2% BSA solution containing anti-sarcomeric myosin antibody MF-20 conjugated with Alexa Fluor 488 (eBioscience). After myonuclei were stained with DAPI (Molecular Probes), micrographs were acquired under a fluorescence microscope (Carl Zeiss, Axio Observer). The cells with a minimum number of three myonuclei were counted as differentiated myotubes. A fusion index is a myological indicator showing the degree of myogenic differentiation. The index is calculated as the number of nuclei in myotubes divided by the total number of nuclei counted, which is expressed as a percentage.

### Quantitative real-time PCR

Differentiated myotubes were washed with cold DPBS and lysed with TRIzol reagent. Chloroform was added for the separation of RNA from DNA and protein fractions. RNA fraction was precipitated by the addition of isopropanol, followed by centrifugation at 12,000 g for 8 min at 4°C. The RNA pellet was washed with 75% ethanol and centrifuged at 12,000 g for 5 min at 4°C. After removal of ethanol, RNA pellets were air-dried before resuspending with RNAse free water. The RNA concentration was quantified using a NanoDrop spectrophotometer (ND-2000, Thermo Fisher Scientific, USA). Reverse transcription was performed with a cDNA synthesis kit according to the manufacturer’s instruction (Bioneer, Korea). Following complementary DNA synthesis, qRT-PCR was performed in 7500 Real-Time PCR system (Applied Biosystems, USA) using a SYBR Green Master Mix (Bioline, Korea), and the primer pair sets are described in Table 1. Cycle threshold (Ct) values were normalized to the housekeeping gene (HPRT1-F, 5'-GACTTGCTCGAGATGTCATG-3'; HPRT1-R, 5'-TACAGTCATAGGAATGGACC-3').

**Table 1.** Primer sets for qRT-PCR

Gene		Primer Sequence (5’ to 3’)
Pgc-1α	Forward	TGATGTGAATGACTTGGATACAGACA
	Reverse	GCTCATTGTTGTACTGGTTGGATATG
Ncor1	Forward	GACCCGAGGGAAGACTACCATT
	Reverse	ATCCTTGTCCGAGGCAATTTG
Nrf1	Forward	GAACGCCACCGATTTCACTGTC
	Reverse	CCCTACCACCCACGAATCTGG
Tfam	Forward	CTGATGGGTATGGAGAAGGAGG
	Reverse	CCAACTTCAGCCATCTGCTCTTC
HPRT	Forward	GACTTGCTCGAGATGTCATG
	Reverse	TACAGTCATAGGAATGGACC

### Immunoblotting

Immunoblotting was performed as described previously^19^. Briefly, differentiated myotubes were lysed in RIPA buffer (10 mM Tris-HCl, 5 mM EDTA, 150 mM NaCl, 1% Triton X-100, 0.1% SDS, 1% deoxycholate, pH 7.5) with cell scraper. Following centrifugation (16,000 g for 15 min at 4°C), supernatants were collected for Bradford assay to quantify total protein concentrations. Following SDS-PAGE, samples were transferred to the PVDF membrane. Subsequently, the membranes were incubated in TBST containing 5% nonfat dry milk for 30 min prior to overnight incubation with respective primary antibodies (rabbit polyclonal anti-VDAC1/porin [Abcam] and mouse monoclonal α-tubulin [Sigma-Aldrich]). After washing twice in TBST, the membranes were further incubated with HRP-conjugated secondary antibodies for 40 min, followed by washing thrice with TBST. Then, membranes were subjected to standard enhanced chemiluminescence (Thermo Fisher Scientific) method for visualization. The resulting band intensities were quantified by ImageJ software (NIH).

### Statistical analysis

The results were presented as mean + SEM for a minimum of six independent experiments. One-way ANOVA was followed by Tukey’s post hoc test to present statistical difference among groups. The statistical significance was set at P-value < 0.05.

## RESULTS

### Effects of L-carnitine and oleate on proliferation and viability of myoblasts

**Fig. 2.**
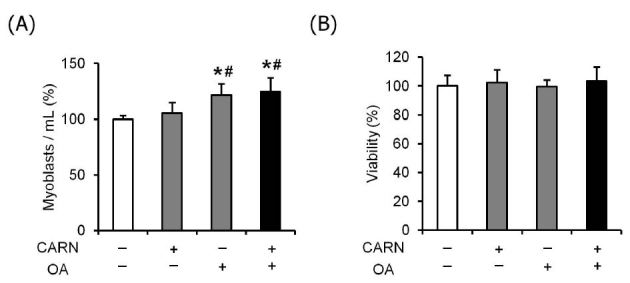
The effects of oleate and L-carnitine on myoblast proliferation and viability. (A and B) Proliferating myoblasts (5 × 104/ml) were seeded onto non-coated 6-well culture dishes and incubated for 48 h in DMEM (10% FBS) supplemented with 300 μM oleate and/or 5 mM L-carnitine. The cells were trypsinized and stained with trypan blue. Non-stained viable cells were counted using a hemocytometer and expressed as %NT. The bar graph represents mean ± SEM. The data were analyzed using one-way ANOVA followed by Tukey’s post hoc test. *p < 0.05 vs non-treated cells; #p < 0.05 vs L-carnitine.

To investigate whether L-carnitine and oleate have effects on cell proliferation and viability, myoblasts were incubated at 37°C for 48 h until the assay. Although no effect on cell proliferation was observed with L-carnitine, the cell number was significantly higher in myoblasts conditioned with oleate (24%) or a mixture (27%) than that in non-treated myoblasts (*P* < 0.05, Figure 2A). Cell viability was not different among all tested groups (Figure 2B).

### Effects of L-carnitine and oleate on myogenic differentiation

**Fig. 3.**
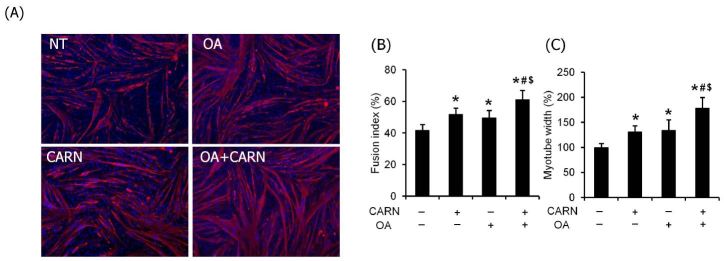
The effects of oleate and L-carnitine on the fusion index and myotube size. (A) Differentiated myotubes were stained with DAPI and MF20 antibody and visualized by florescence microscopy (magnification = 20×). (B) Fusion index and (C) myotube width were quantified and expressed as %NT. The bar graph represents mean ± SEM. The data were analyzed using one-way ANOVA followed by Tukey’s post hoc test. *p < 0.05 vs non-treated cells; #p < 0.05 vs L-carnitine, $p < 0.05 vs oleate.

To investigate the effects of L-carnitine and oleate on myogenic differentiation, the growth medium without L-carnitine and oleate was used for incubation until 95% confluency. After proliferation, the growth medium was changed to the respective DM. The morphological assay indicated that fusion index and myotube width were significantly higher in myotubes conditioned with L-carnitine or oleate than those of non-treated myotubes (*P* < 0.05). Interestingly, the values of the fusion index and myotube width were significantly higher in myotubes conditioned with a mixture (*P* < 0.05) in contrast to those with L-carnitine or oleate alone (Figure 3A–C).

### Effects of L-carnitine and oleate on mitochondrial biogenic gene expressions

**Fig. 4.**
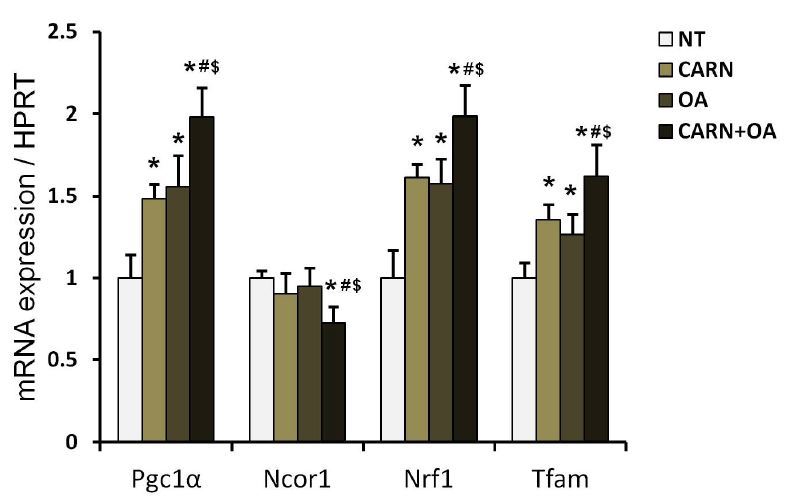
The effects of oleate and L-carnitine on mRNA expression levels during mitochondrial biogenesis in differentiated myotubes. The gene expressions of Pgc1-α, Ncor1, Nrf1, and Tfam were quantified by qRT-PCR. The results were normalized by HPRT expressions. The bar graph represents mean ± SEM. The data were analyzed using one-way ANOVA followed by Tukey’s post hoc test. *p < 0.05 vs non-treated cells; #p < 0.05 vs L-carnitine; $p < 0.05 vs oleate.

As the mitochondrial biogenic process is coupled with initiation of myogenic differentiation, the related gene expressions were measured following differentiation for 96 h. The expressions of deacetylation of peroxisome proliferator- activated receptor-γ coactivator 1-alpha (Pgc1-α), nuclear respiratory factor (Nrf1), and mitochondrial transcription factor A (Tfam) were increased in myotubes conditioned with L-carnitine and oleate, individually, in contrast to those with no treatment, with a greater effect observed in myotubes conditioned with the mixture (P < 0.05, Figure 4). The expression of Ncor1, a corepressor and reciprocal regulator of Pgc1-α, was not changed by individual treatment of L-carnitine and oleate, whereas this gene expression was decreased significantly in myotubes conditioned with a mixture of L-carnitine and oleate than that in non-treated myotubes (*P* < 0.05, Figure 4).

### Effects of L-carnitine and oleate on protein contents of porin

**Fig. 5.**
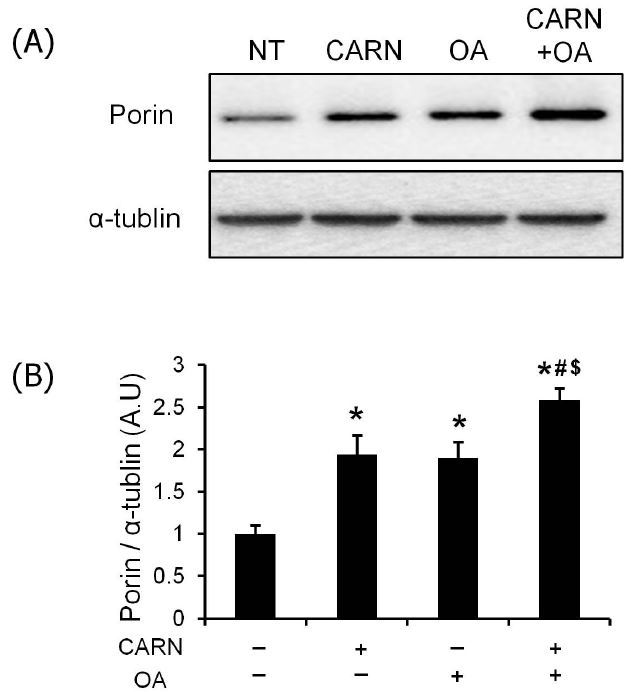
The effects of oleate and L-carnitine on protein level of porin. Immunoblotting was performed to measure the protein expression of mitochondrial mass surrogate marker. (A) The images of porin were quantified by NIH ImageJ software. (B) The loading volume was normalized by the protein amount of α-tubulin. The bar graph represents mean ± SEM. The data were analyzed using one-way ANOVA followed by Tukey’s post hoc test. *p < 0.05 vs non-treated cells; #p < 0.05 vs L-carnitine; $p < 0.05 vs oleate.

The protein level of porin was measured in differentiated myotubes as a surrogate marker of mitochondrial mass. The protein expression of porin was significantly increased by individual treatment of L-carnitine and oleate than that in non-treated myotubes (*P* < 0.05, Figure 5). The protein levels of porin were significantly higher in myotubes conditioned with a mixture in contrast to those with L-carnitine or oleate alone (*P* < 0.05, Figure 5)

## DISCUSSION

Although myogenic progenitors have innate capabilities to mediate myofiber regeneration, development of an efficient strategy is warranted to reduce the recovery duration of injured myofibers. As the orchestrated regulation of myoblasts and proliferating myogenic progenitors are prerequisite for skeletal muscle regeneration, the effects of L-carnitine and oleate on overall cellular potential of myoblasts were investigated. Here, we demonstrated that the combined treatment of L-carnitine and oleate promotes myoblast proliferation and myogenic differentiation with concomitant increment of mitochondrial mass. This indicates that L-carnitine- and oleate-mediated myogenic differentiation is promoted possibly via modulation of expression of mitochondrial genes.

Myosatellite cells, which are myogenic progenitors, are located in the sublaminar niche in a mitotically quiescent condition^20^. Once activated by injury-induced endogenous and exogenous stimuli, quiescent satellite cells are subjected to a self-renewing process to induce myoblasts proliferation^21^.

In the study, oleate and a mixture of L-carnitine and oleate stimulated the rate of myoblast proliferation after 48 h treatment (Figure 2A). This indicates that the promotion in the proliferation of these cells was independent from the effect of L-carnitine and this phenomenon was mainly derived from the oleate effect. This is in line with a previous study that the treatment of oleic acid alone increased myoblast proliferation^22^. As the oleate-mediated proliferation was not retarded under the mixture condition, it is suggested that the application of oleate or a mixture of oleate and L-carnitine could be considered to promote the initial step of the myogenic progenitor’s self-renewal process during muscle regeneration in the absence of detrimental side effects. This idea was further supported in the viability tests wherein cellular toxicity was not observed in myoblasts conditioned with L-carnitine and/or oleate (Figure 2B).

Satellite cells move along the myofiber in an external chemotactic signaling molecule-dependent manner^23^. As these cells are sparsely scattered along the myofiber, the migration of activated satellite cells is necessary following the self-renewing process^24^. On the microscopic injury site, the migrated myoblasts are elongated for the facilitation of complete regeneration, called myogenic fusion. This process is initiated by early myogenic determination, leading to myogenin-induced differentiation to incorporate into myofibers^20^.

In the study, both L-carnitine and oleate individually increased fusion index (myological indicator) and myotube width. Interestingly, a mixture of both further increased these values over the individual treatment (Figure 3A– C). To the best of our knowledge, this is the first study reporting that L-carnitine and oleate have synergistic effects on myogenic differentiation. In line with our results, multiple studies have demonstrated that the molecular structure of intracellular fatty acids is a critical factor and that it is either conducive or detrimental to skeletal muscle physiology. Although most saturated fatty acids have been proven to induce pathophysiological conditions in skeletal muscles^25,26^, oleate has been demonstrated to preserve the integrity of skeletal muscle fibers via deacetylation of Pgc1-α^27^. As the biological role of L-carnitine is to promote the transportation of long-chain fatty acids across the inner mitochondrial membrane into the matrix for beta-oxidation and induce oxidative phosphorylation in skeletal muscles^28,29^, it is speculated that oleate is used as a major substrate to enhance mitochondrial biogenesis during myogenic differentiation.

Mitochondrion, a cellular energy powerhouse, is a dynamic subcellular organelle modulating their mass in response to endogenous and exogenous stimuli in order to maintain an efficient metabolic capacity^30-32^. In regard to mitochondrial-mediated myogenic differentiation, several lines of studies proved that myogenic differentiation was closely linked with the mitochondrial biogenic and remodeling process^33,34^, suggesting that metabolic shift to oxidative phosphorylation in conjunction with an increase in mitochondrial biogenesis is essential for complete differentiation of myoblasts. Given that the gene expressions of mitochondria-related coactivator (Pgc1-α) and transcription factors (Nrf1, Tfam) were the highest and that of mitochondria-coupled corepressor (Ncor1) was the lowest in the combined treatment condition (Figure 4), it indicates that oleate and L-carnitine promote myogenic differentiation via modulation of mitochondrial biogenic gene expressions in a synergistic manner, as further evidenced by the highest protein level of a surrogate marker of mitochondrial mass (porin) in myotubes conditioned with a mixture (Figure 5).

**Fig. 6.**
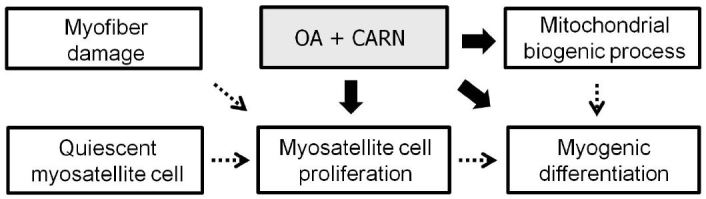
Schematic diagram of a proposed mechanism for myogenic progenitor-mediated myofiber regeneration. The dotted arrows indicate the series of biological processes involved in myofiber regeneration. The filled arrows indicate treatment effects on cellular behavior of myogenic progenitors. OA, oleic acid; CARN, L-carnitine.

In summary, this study demonstrated that oleate increases myoblast proliferation, and the combined treatment of L-carnitine and oleate promotes myogenic differentiation in a synergistic fashion, coupled with the enhanced expression of mitochondrial biogenic genes (Figure 6). These results provide a critical piece of cellular evidence that treatment with oleate and L-carnitine could be considered as an effective therapeutic strategy to activate myogenic progenitors for the regeneration of myofibers. In future endeavors, optimal timing and intake dose of these nutrients should be investigated, which warrants future ex vivo and in vivo studies for the development of novel nutritional strategies to ameliorate skeletal muscle injury.
